# A mixture-density-based tandem optimization network for on-demand inverse design of thin-film high reflectors

**DOI:** 10.1515/nanoph-2021-0392

**Published:** 2021-10-08

**Authors:** Rohit Unni, Kan Yao, Xizewen Han, Mingyuan Zhou, Yuebing Zheng

**Affiliations:** Walker Department of Mechanical Engineering, The University of Texas at Austin, Austin, TX 78712, USA; Texas Materials Institute, The University of Texas at Austin, Austin, TX 78712, USA; Department of Statistics and Data Science, The University of Texas at Austin, Austin, TX 78712, USA; McCombs School of Business, The University of Texas at Austin, Austin, TX 78712, USA

**Keywords:** artificial neural networks, deep learning, inverse design, nanophotonics, optimization, thin-film optics

## Abstract

Deep learning (DL) has emerged as a promising tool for photonic inverse design. Nevertheless, despite the initial success in retrieving spectra of modest complexity with nearly instantaneous readout, DL-assisted design methods often underperform in accuracy compared with advanced optimization techniques and have not proven competitive in handling spectra of practical usefulness. Here, we introduce a tandem optimization model that combines a mixture density network (MDN) and a fully connected (FC) network to inversely design practical thin-film high reflectors. The multimodal nature of the MDN gives access to infinite candidate designs described by probability distributions, which are iteratively sampled and evaluated by the FC network to allow for rapid optimization. We show that the proposed model can retrieve the reflectance spectra of 20-layer thin-film structures. More interestingly, it reproduces with high precision the periodic structures of high reflectors derived from physical principles, even though no such information is included in the training data. Improved designs with extended high-reflectance zones are also demonstrated. Our approach combines the high-efficiency advantage of DL with the optimization-enabled performance improvement, enabling efficient and on-demand inverse design for practical applications.

## 1 Introduction

Deep learning (DL) has emerged as a promising and powerful tool for many tasks in optics and photonics, such as image reconstruction and enhancement in microscopy [1], feature recognition in spectroscopy [2], and inverse design of photonic devices [3], [4], [5], [6], [7], [8], to name a few. Conceptually, DL uses many-layered neural networks (NNs), where a series of parameters are adjusted upon exposure to a repository of training data, to learn an abstract and generalizable mapping from the inputs to the outputs of NNs. In the case of inverse design, the inputs are the desired optical responses, and the outputs are the design variables of a coinciding structure. Owing to their remarkable ability to unearth unintuitive and hidden relations within the data, DL-based methods have been applied to designing various photonic structures, including metasurfaces [9], [10], [11], metagratings [12, 13], and multilayer nanoparticles and thin films [14], [15], [16], [17], [18], [19], [20], [21], etc. The choice of the assisting NNs also diversifies quickly from fully connected (FC) networks to many advanced models (discriminative or generative) and their ensembles [22], [23], [24], [25]. While these efforts have shown encouraging ability to reproduce spectra based on fully random designs or simple physical processes such as a Lorentzian resonance, little has been reported at the level of solving practical problems, for which some rules of design have been derived from physical principles. Thus, DL has yet to convincingly show a capacity to match or replace traditional physics-based design methods. In addition, in comparing with optimization techniques for inverse design [26], [27], [28], DL offers an unparalleled advantage of high speed after training but is less favorable in accuracy, especially when the target optical responses contain steep features resulting from sophisticated physical processes. Recent works have shown the potential of incorporating both DL and optimization in inverse design [10, 14, 29], which could combine the advantages of high speed and scalability from DL with the higher maximum performance afforded by optimization techniques. This attempt raises the possibility that the most fruitful avenue for application of DL to inverse design may be in concert with existing approaches.

**Figure 1:**
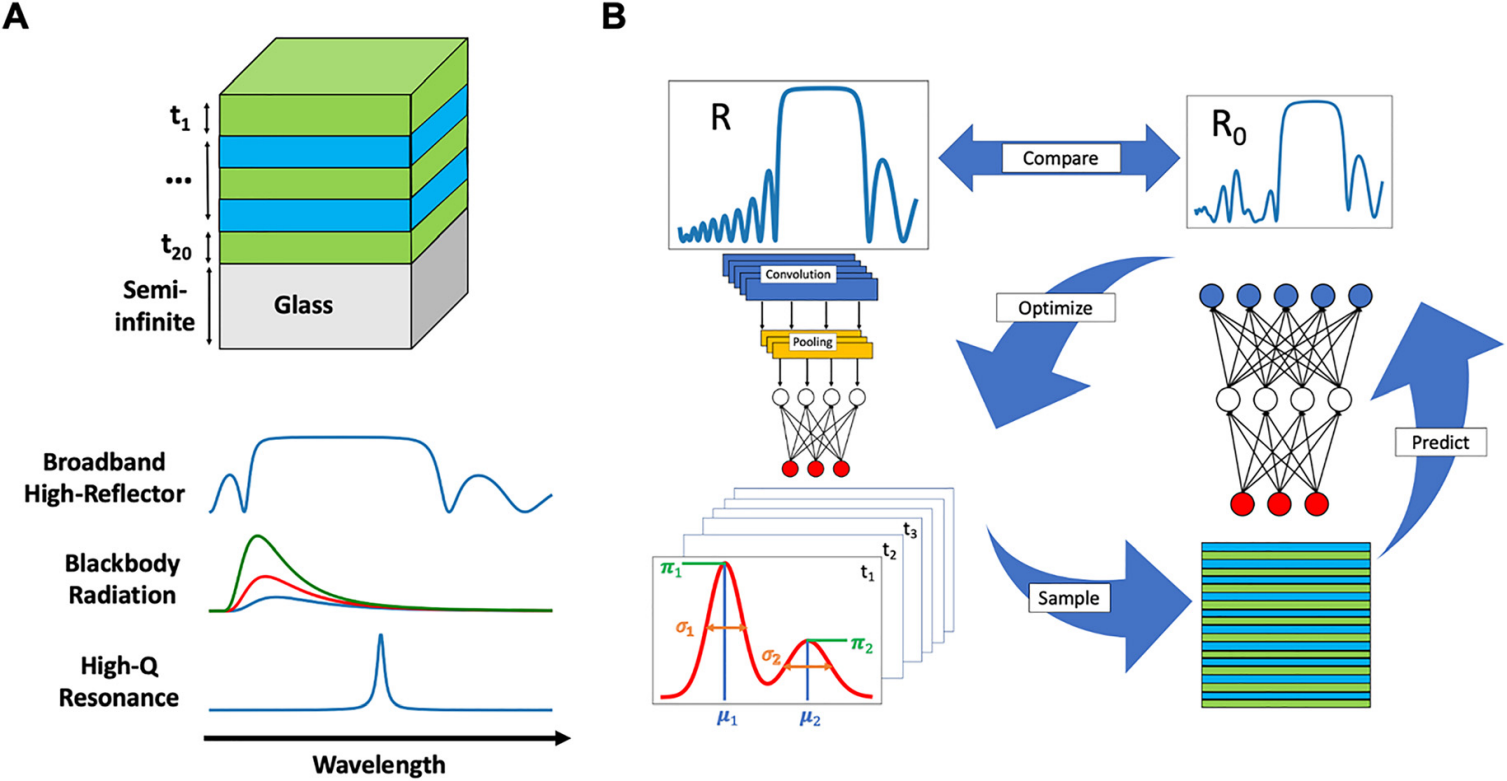
(A) Top: diagram of a 20-layer thin-film structure consisting of two alternating materials placed on a semi-infinite glass substrate. Once the materials are chosen, the thicknesses of layers *t*_1_ – *t*_20_ constitute the design parameters for any requested optical responses. Bottom: examples of optical spectra of the multilayer thin-film structure featuring selected line shapes for various applications, including broadband high reflection, black body radiation, and narrow peaks from resonances. (B) Schematic of the tandem model with adjoining optimization method. The requested or target spectrum *R* (navy curve) fed into the MDN (left column) produces probability distributions of the design parameters (red curve), which are repeatedly sampled and evaluated with the forward model (right column) to optimize the designs until the predicted optical response *R*_0_ (blue curve) is close enough to the target spectrum *R*.

In this work, we report a tandem model that combines two NNs with an optimization process to inversely design a type of multilayer thin-film structure with wide applications, which are known as the high reflectors. Among the structures to which DL-based design methods have been applied, multilayer stacks are of particular interest [15], [16], [17], [18, 30]. On one hand, thorough understanding of the properties of multilayer structures sets the basis of thin-film optics. Albeit the seemingly simple geometry, multilayer thin films can provide a variety of optical properties (see Figure 1A), depending on the choice of materials and their arrangements. The associated devices span beam splitters [31], high reflectors [32], antireflection coatings [33], and numerous optical filters [34]. In different application scenarios, the exact line shape of the desired spectra is case-specific, each corresponding to a structure to be inversely solved. Practical thin-film devices can have tens or hundreds of layers, making the task challenging enough to stimulate innovative design methods. On the other hand, the development of thin-film optics has reaped many classical designs based on physical principles or rules of thumb [34]. Retrieving and outperforming these devices can be good gauges to assess the performance of numerical design methods.

Our two NNs include a mixture density network (MDN), which solves the inverse problem to suggest probability distributions of “raw” designs [35], and a standard FC network as a forward simulator to instantly evaluate any updated design during iterative optimization. The applicability of the proposed model is beyond retrieval of random thin-film structures. We show that the classical design of high reflectors, derived from the principle of interference of light, can be reconstructed with high precision, even though no information about the periodicity or quarter-wavelength layer thickness is introduced into the training data. Improved designs with extended high-reflectance regions, which are not easily obtainable by physics-based design rules, are also demonstrated. We emphasize that the use of two NNs in this work differs from previous implementations of a tandem architecture [15, 36], which feature the inverse and forward networks directly connected to relieve non-uniqueness. Here, the non-uniqueness is relieved by the MDN alone, and the forward model is indirectly connected via the optimization method to iteratively improve designs after initial readout. The model is also different from recent works that use NNs only to accelerate the adjoint simulations in an optimization framework [30, 37]. In fact, apart from resampling, our whole model is DL-based. The unique ability of MDN to predict multiple optima of the objective function can potentially aid in the search of a global optimum rather than getting stuck at a local one. Therefore, our approach combines the advantage of DL in high speed with the optimization-enabled performance improvement, enabling efficient and on-demand inverse design of structures and devices for practical applications.

## 2 Materials and methods

As illustrated in Figure 1B, we construct a tandem model consisting of two independent deep neural networks. The first network is an MDN, designated to solve the inverse problem, i.e., seeking a design that will produce the desired optical properties. The unique feature of MDNs is that the outputs are not deterministic discrete design variables but their probability distributions over the possible ranges. These distributions are built as a mixture of *M* (we take *M* = 16 in this work) Gaussian distributions, each parametrized by a mean *μ*, variance *σ* and a mixing weight *π*, with the mixing weights shared across all design mixtures. These parameters are what the network outputs in lieu of directly outputting design values. Candidate designs are then generated by sampling the distributions of the individual variables. To train the model, desired outputs are measured for goodness of fit with the model-produced distributions using a metric such as log-likelihood, and this allows for multiple correct outputs to be modeled as different peaks in the distribution. This difference from ordinary NNs alleviates the non-uniqueness problem in inverse design, and more importantly, opens the opportunity of subsequent optimization for performance improvement and better search of a global optimum in the design space. The second network deals with the forward problem, functioning as a simulator to predict the optical response of a given structure. This task is relatively simple and can thus be carried out by a standard FC network. A full description of the model architectures, hyperparameters, and training curves is given in Section 1 of the Supplementary materials. Upon completion of training, both the suggestion of candidate designs for a given spectrum and the prediction of optical properties for a given design are almost instantaneous.

To work jointly as a tandem, the two NNs are connected through an optimization procedure, which essentially post-processes the output of the MDN by iteratively evaluating and updating sampled candidate designs with the assistance of the forward network. The full model works as follows for a design task involving *N* variables. First, a desired optical spectrum *R* is fed into the MDN, which produces at the output *N* probability distributions, each corresponding to one design variable. Without complex sampling strategies, the initial guess of the design is made by assigning every variable the value at the most prominent peak of its own distribution. Next, the initial design suggestion is sent to the forward network for a prediction of the optical response, denoted by *R*_*M*_ and also labeled as *R*_0_ to indicate that it is the predicted response of the active candidate design. The performance of this design is evaluated by comparing *R*_0_ with the ground truth *R* based on some metrics, including root mean square error (RMSE). Then, the optimization process starts. Of the *N* variables of the active candidate design, a randomly chosen one is resampled for a specified number of times based on its probability distribution for new guesses, while the remaining *N* − 1 variables are fixed. Once the predicted response of a new guess *R*_*i*_ is closer to *R* than *R*_0_, that guess becomes the new active candidate design, and *R*_0_ is updated accordingly. The resampling and evaluation repeat for all the *N* variables in a random order, and the process of cycling through all the variables can also be repeated for any number of times. A more detailed account on the numbers chosen for optimization and their relation to improvement in the design is provided in Section 2 of the Supplementary materials. If the forward model is accurate enough, the prediction of the design guesses’ error relative to the ground truth will closely approximate the true error, and the design will improve over time. Lastly, after the optimization is complete, the optical response of the finalized design *R′* is simulated by plugging the design variables in an electromagnetic solver, which computes the real properties of the final design. It should be emphasized that through the entire design process, simulation is only used once at the very end for verification.

We apply our model to inverse design based on the reflectance from a stack of dielectric thin films of alternating high and low refractive indices. The whole structure consists of 20 layers of magnesium fluoride (MgF_2_) and tantalum pentoxide (Ta_2_O_5_) placed on a glass substrate and with an air cladding, as shown in Figure 1A. For the sake of comparison to physics-based design rules, only normal incidence is considered. This reduces the design variables to the thicknesses of each of the 20 layers, forming a 20-dimensional vector. However, inclusion of the angle of incidence in the design variables has no intrinsic difficulty [16]. The reflectance is calculated for the wavelength interval of 400–1000 nm using analytical formulae based on the Fresnel equations [38], and the spectrum is discretized into 300 points. We limit the ranges of possible physical thicknesses to between 50 and 150 nm for MgF_2_ and between 30 and 120 nm for Ta_2_O_5_, both of which cover the optical thickness of quarter-wavelength for a large portion of the wavelengths of interest. The dielectric functions of MgF_2_ and Ta_2_O_5_ are taken from Refs. [39, 40] unless otherwise specified. To train the two separate models, we use the same dataset of 828,000 samples, split into 70% for training and the remaining 30% for testing. In sample generation, 50% of the data uniformly samples the entire thickness range for all design variables, 25% of the data uniformly samples the upper half of the thickness range for each, and the last 25% uniformly samples the lower half. The uniform sampling means all values within the chosen interval are equally likely to be chosen. Other sampling techniques such as low-discrepancy sequences [41] are also tested and produce qualitatively similar results, as discussed in the Supplementary materials, Section 3. Compared with sampling the full thickness ranges all at once, this strategy ensures that structures with relatively balanced thicknesses are less likely underrepresented, given the enormous design space spanned by 20 independent variables.

## 3 Results

Both models are trained via gradient descent for 500 epochs on the Stampede2 computer of Texas Advanced Computing Center (TACC). A single compute node is used for training, which takes a total of approximately 23 h to complete, in addition to about 1 h for data generation. The forward model uses RMSE in the optical response as its loss function and converges to a final RMSE of 0.02 for both the training and test datasets. The MDN uses the negative log-likelihood metric and converges to a value of −18 for both datasets. The optimization process is set to run four cycles of resampling and evaluation through all the 20 variables, and each variable is sampled 50 times in one cycle. With these settings, the optimization of a single design takes approximately 1 min on the same TACC compute node or 6 min on a commercial laptop.

### 3.1 Retrieval of random stacks

**Figure 2:**
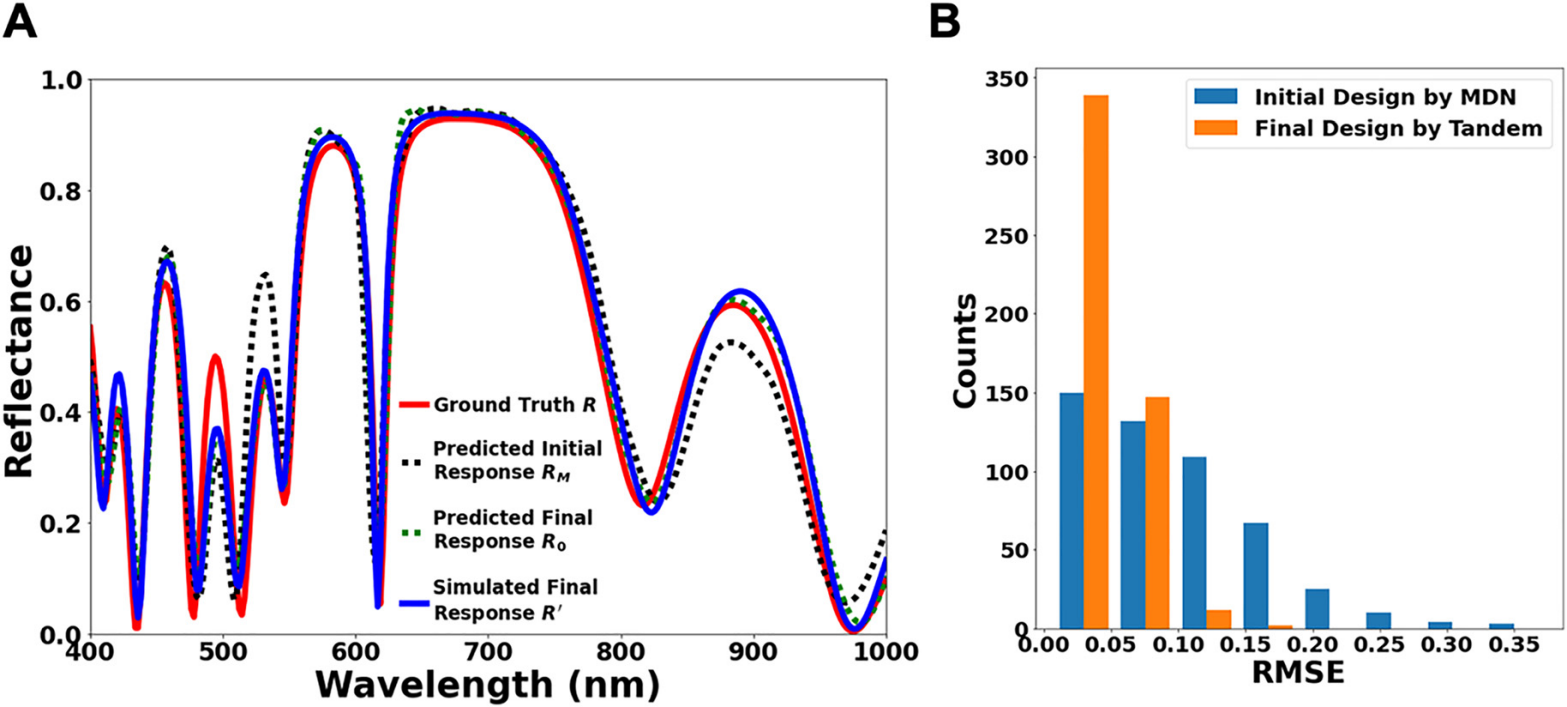
(A) Comparison of a requested spectrum *R* (red curve) with the simulated response *R′* of the final design retrieved by the model (blue curve) for a random case from the test dataset. Forward model predictions of the response of the initial MDN design *R*_*M*_ (black dash curve) and of the final design *R*_0_ (green dash curve) are also shown. (B) Histogram of RMSE matching between the requested spectrum and the response of the model-suggested design for 500 randomly chosen test dataset samples before optimization (initial design from the MDN) and after optimization (final design by the tandem optimization model).

We first examine the tandem optimization model on the spectra produced by random structures in the test dataset. The model can retrieve those designs fairly well even with the MDN alone, and progressive improvements can be seen during the optimization process. Figure 2A presents an example randomly chosen from the test dataset. Three spectra taken at different steps of the design, namely the predicted response of MDN’s initial guess *R*_*M*_ (black dash curve), the predicted response of the final design *R*_0_ (green dash curve), and the simulated response of the final design *R’* (blue curve), are compared with the desired spectrum as ground truth *R* (red curve). Noticeably, despite the complex line shape of the target spectrum, the initial guess that simply takes the peak values of the outputs of MDN can already resemble the ground truth design closely at most wavelengths. The deviations between the initial guess and the ground truth are diminished by optimization. Both the predicted and simulated responses of the final design overlap the target spectrum *R* almost perfectly, confirming the effectiveness of the optimization procedure and the accuracy of the forward network. To get a quantitative idea of the performance of MDN and the improvement by optimization, we conduct a statistical study over 500 samples randomly chosen from the test dataset. Figure 2B shows the histograms of RMSE between the requested spectra and the responses of the initial guesses by MDN and final designs after optimization. The initial designs, sampled at the most prominent probability peaks predicted by the MDN, give an average response RMSE of 0.09. In comparison, after optimization, the model produces final designs with an average RMSE of 0.04, improved by more than a factor of two. This improvement highlights the unique benefits of the probabilistic nature of the MDN. By resampling and using the forward model to evaluate samples without additional simulations, designs can be continuously optimized after an initial sample. The optimization can run for any arbitrary number of rounds and samples, with the processing time scaling linearly with each factor, however the improvements in response RMSE see diminishing returns. A more detailed description is provided in Section 2 of the Supplementary materials.

### 3.2 Retrieval of classical high reflectors

**Figure 3:**
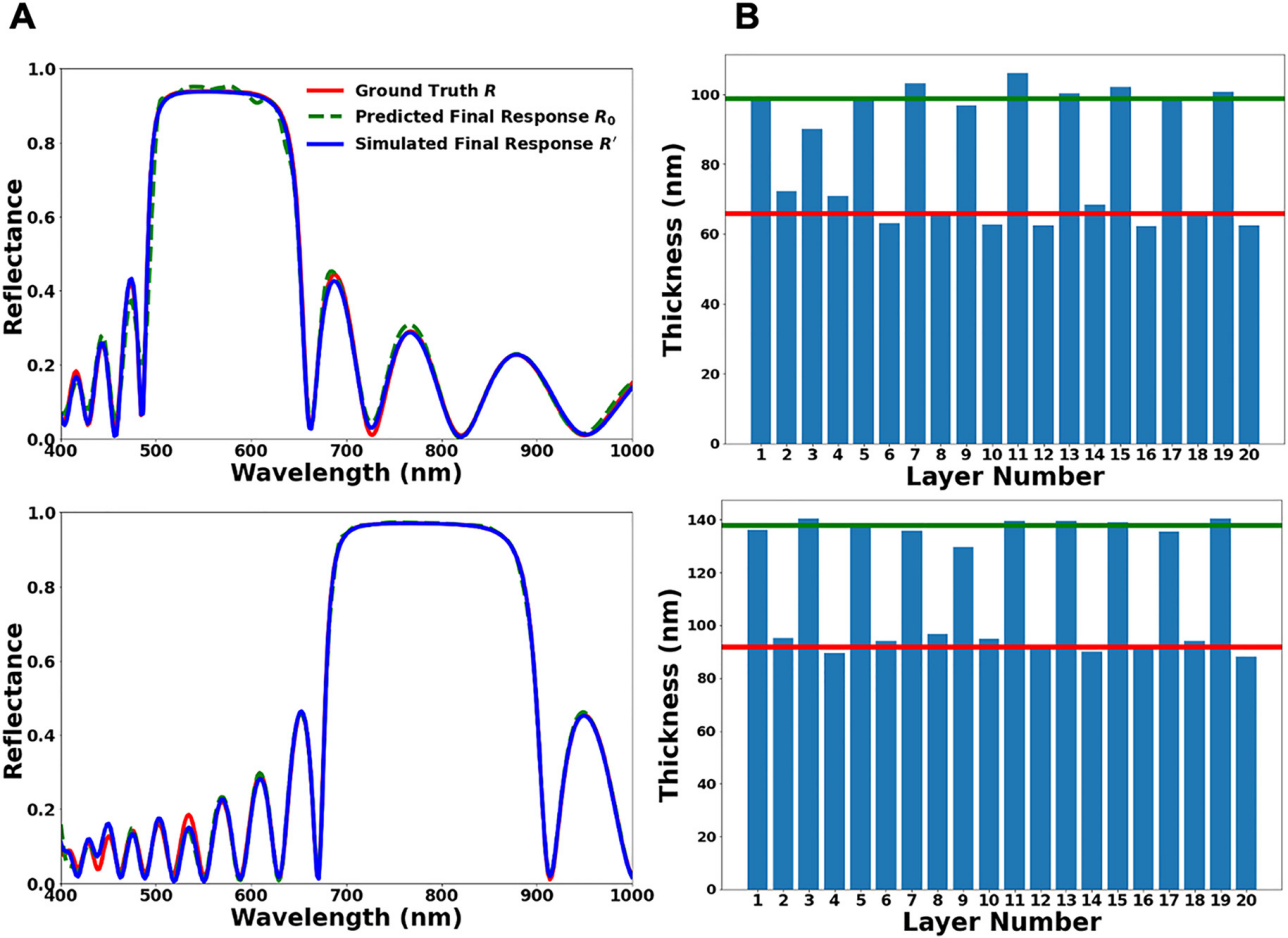
Inverse design of DBRs consisting of alternating high- and low-index films of quarter-wavelength thicknesses stacked in a periodic fashion. (A) Comparisons of the requested spectrum *R* from a DBR (red curve) with the simulated response *R′* of the final design retrieved by our model (blue curve). Also shown is the predicted response *R*_0_ of the final design (green dash curve). Two examples are selected for shorter (top) and longer (bottom) central wavelengths. (B) Visualization of the 20-layer thicknesses for the corresponding designs (top: shorter central wavelength; bottom: longer central wavelength) retrieved by the tandem optimization model for both requested spectra. Green and red lines mark the alternating thicknesses of MgF_2_ and Ta_2_O_5_, respectively, in the ground truth designs of DBRs.

Practical applications of the multilayer thin films usually require optical responses that are much more sophisticated than a series of slowly varying peaks. Typical features like a constant response over a certain wavelength range and steep edges cannot be produced by randomly assembled layers as building blocks. Therefore, the ability to retrieve spectra from random structures is no guarantee of practical applications. To prove that the proposed model can be a competitive tool to tackle real-world applications, we further test it with a classical type of thin-film high reflectors, the distributed Bragg reflectors (DBRs) [42, 43], which feature a flat high-reflectance band and are originally accessed by physics-based approaches. The most popular design of DBRs consists of a stack of quarter-wave dielectric films with alternating high and low refractive indices. In other words, a DBR has a periodic structure, and with a given pair of materials, the thicknesses of neighboring layers are determined by the central wavelength of operation. We compute the reflectance of several DBRs targeting at different wavelengths and feed the spectra into the MDN as the desired optical properties. The proposed model performs very well in retrieving these DBR designs, as shown in Figure 3. In both cases at shorter and longer wavelengths, the predicted and simulated responses of the final designs are highly coincident with the ground truth spectra. More impressively, the model manages to “discover” periodicity in the appearance of two materials, and the suggested thicknesses only deviate slightly from the quarter-wavelengths. With a total of 20 independent design variables, the size of our dataset gives an extremely low chance of any individual training samples being periodic to supply this knowledge to the model. Therefore, the successful retrieval of DBRs, despite their significant divergence from the random structures used for training, suggests that the model has a strong ability to intuit the design-response mapping on a fundamental level.

The working principle of DBRs is the constructive interference of light fields reflected from each interface of the multilayer thin films, while a random arrangement of thicknesses cannot lead to such an effect. We note two interesting phenomena regarding this difference. One is that DBRs are tolerant of small disturbances to the ideal design. In Figure 3B, the deviations of the retrieved thicknesses from ground truths are largely attributed to this tolerance. The other is that (quasi-)periodic structures may apply implicit constraints that suppress multimodality in the design. The final thicknesses after optimization in Figure 3 are close to those taken from the MDN’s initial suggestion. In contrast, randomly sampled designs have a much greater chance to contain at least one layer that exhibits secondary peaks in the corresponding probability distribution. A brief discussion of multimodality is presented in Section 3 of the Supplementary materials.

### 3.3 Inverse design of reflectors with extended high-reflectance zones

**Figure 4:**
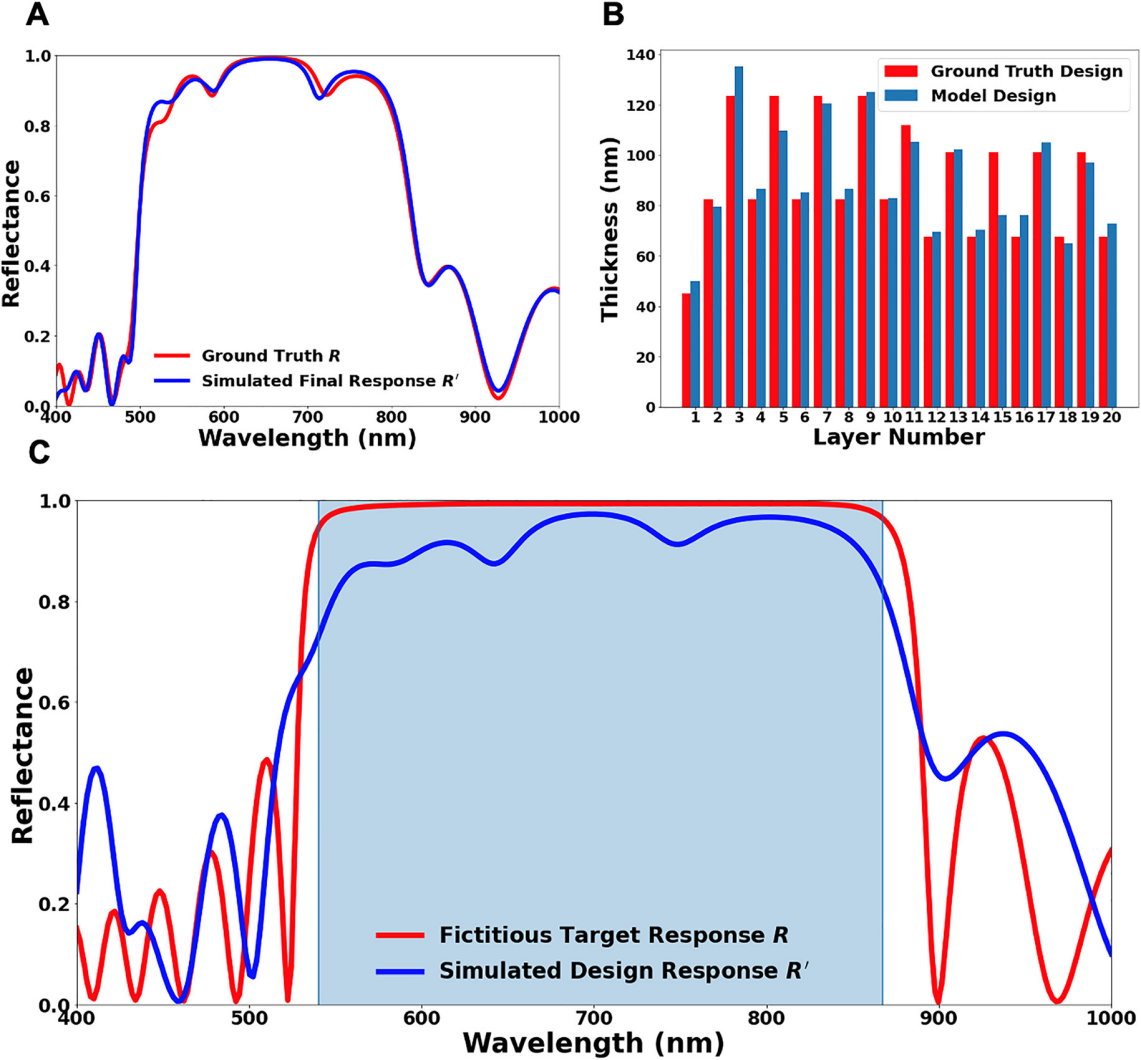
On-demand inverse design of thin-film high reflectors with extended high-reflectance zones. (A) Comparison of a requested spectrum *R* and the simulated response *R′* of the design retrieved by the model. The target design is composed of two 9-layer periodic sub-stacks with a spacer layer in the middle and a cladding layer on the top, obtained with a known optimization strategy based on the principle of interference. (B) Comparison of design variables between the ground truth design and model-produced design. (C) Inverse design of a high reflector with ultrabroad high-reflectance zone. The requested spectrum *R* (red curve) is fictitious, taken from a 20-layer DBR and artificially extended in the high-reflectance region for a bandwidth not achievable by known design methods based on physical principles. The blue curve shows the simulated response *R′* of the design suggested by the tandem optimization model. Note that in this case, optimization is applied to the high-reflectance region only, as highlighted by the shaded area.

Our next task is set to be more ambitious. Standard DBRs have a limited bandwidth, determined by the choice of materials. In the frequency domain, the width of the high-reflectance zone ∆*f* is given by [34]

(1) $$\frac{\Delta f}{f_{0}}=\frac{4}{\pi }\arcsin\left(\frac{n_{H}-n_{L}}{n_{H}+n_{L}}\right)\text{,}$$

where *f*_0_ is the central frequency, and *n*_*H*_ and *n*_*L*_ are the refractive indices of the high and low index layers, respectively. Extending the width of the high-reflectance zone is a task of practical concern, and different attempts have been made based on physical considerations or optimization algorithms [34]. We first challenge our model with a design manually optimized with the guidance of interference conditions, which requests stacking two 9-layer periodic structures covering different wavelengths with a spacing layer in the middle and a cladding layer on the top. Again, the model retrieves the design with high precision. To minimize the influence of the dielectric loss, we train the model with a different dataset generated using the dielectric function of Ta_2_O_5_ measured by Gao et al. [44]. Figure 4A compares the performance of the final design and the ground truth, showing an excellent agreement. The small dips in the extended high-reflectance zone are unavoidable mainly because of the limited number of layers. Figure 4B shows the comparison of design variables. The two distinct sub-stacks can be clearly recognized, despite that no such information is provided in the training data. With only nine layers in each sub-stack, the extraction of strict periodicity is more difficult than in the standard DBRs.

Finally, we solve the ultimate task to search for high-reflector designs that can outperform the examples derived from known principles. Although any fictious spectra can be used as the input to the model, we choose to artificially extend the high-reflectance zone of a DBR to be wider than what is achievable by the previous optimization method [34]. This manipulation creates a spectrum in Figure 4C (red curve), which does not coincide with any known structure and may not be physically possible with the constraints of the design space. However, the model successfully finds a design with consistent high reflectance across the desired wavelength range (blue curve). In this specific task, the optimization process is customized to evaluate the candidate designs only in the target high-reflectance zone. The flexibility of tuning the objective function of optimization further enhances the search for the closest possible matching design to any input spectra.

## 4 Conclusions and future study

We propose a framework for inverse design based on two NNs combined through an optimization process and demonstrate its ability to outperform physics-based methods in designing thin-film high reflectors. The first NN solving the inverse problem is the MDN. Its unique probabilistic nature allows generation of candidate designs with information of uncertainty, which enables progressive performance improvement through iterative evaluation and resampling during the optimization process. The second NN has a standard FC architecture to solve the forward problem. It serves as a simulator to make instant and accurate predictions of the response of candidate designs from the MDN. We apply this tandem optimization model to on-demand inverse design of high reflectors based on 20-layer thin films. In addition to passing the ordinary test of reproducing the reflectance spectra of random structures, the model successfully retrieves a series of high reflector designs that are derived by physics-based methods. In particular, DBRs with periodic quarter-wave layers are obtained with high accuracy, even though no prior knowledge of periodicity is in the training data. We further demonstrate designs with extended high-reflectance zones, originally accessible only by physical principles or other optimization techniques. Lastly, the model shows a strong ability to search for the closest possible solution to unrealistic reflectors with an artificially widened high-reflectance zone. The proposed model can both quickly and repeatedly produce high-performance designs that are competitive with those obtained by other time-consuming inverse design approaches, making it a promising tool for on-demand industrial applications.

It is worth mentioning that the individual components of the proposed model are highly adaptable. For example, the MDN can be replaced by generative NNs to tackle pattern-based designs, and the optimization would then take samples in the latent space instead of the probability distributions [10]. The success in retrieving periodic structures of DBRs is also encouraging for other uses beyond inverse design, such as knowledge discovery [45], [46], [47], [48], [49]. We envision that the combination of DL algorithms and optimization techniques will provide new opportunities for advancing both optical physics and engineering applications.

## Supplementary Material

Supplementary Material

## References

[1] de Haan K., Rivenson Y., Wu Y., Ozcan A. (2020). Deep-learning-based image reconstruction and enhancement in optical microscopy. *Proc. IEEE*.

[2] Ho C.-S., Jean N., Hogan C. A., Blackmon L., Jeffrey S. S., Holodniy M. (2019). Rapid identification of pathogenic bacteria using Raman spectroscopy and deep learning. *Nat. Commun.*.

[3] Yao K., Unni R., Zheng Y. (2019). Intelligent nanophotonics: merging photonics and artificial intelligence at the nanoscale. *Nanophotonics*.

[4] Hegde R. S. (2020). Deep learning: a new tool for photonic nanostructure design. *Nanoscale Adv.*.

[5] Jiang J., Chen M., Fan J. A. (2021). Deep neural networks for the evaluation and design of photonic devices. *Nat. Rev. Mater.*.

[6] Ma W., Liu Z., Kudyshev Z. A., Boltasseva A., Cai W., Liu Y. (2021). Deep learning for the design of photonic structures. *Nat. Photonics*.

[7] Wiecha P. R., Arbouet A., Girard C., Muskens O. L. (2021). Deep learning in nano-photonics: inverse design and beyond. *Photonics Res.*.

[8] So S., Badloe T., Noh J., Bravo-Abad J., Rho J. (2020). Deep learning enabled inverse design in nanophotonics. *Nanophotonics*.

[9] Liu Z., Zhu D., Rodrigues S. P., Lee K. T., Cai W. (2018). Generative model for the inverse design of metasurfaces. *Nano Lett.*.

[10] Kudyshev Z. A., Kildishev A. V., Shalaev V. M., Boltasseva A. (2020). Machine-learning-assisted metasurface design for high-efficiency thermal emitter optimization. *Appl. Phys. Rev.*.

[11] Nadell C. C., Huang B., Malof J. M., Padilla W. J. (2019). Deep learning for accelerated all-dielectric metasurface design. *Opt. Express*.

[12] Jiang J., Sell D., Hoyer S., Hickey J., Yang J., Fan J. A. (2019). Free-form diffractive metagrating design based on generative adversarial networks. *ACS Nano*.

[13] Inampudi S., Mosallaei H. (2018). Neural network based design of metagratings. *Appl. Phys. Lett.*.

[14] Peurifoy J., Shen Y., Jing L., Yang Y., Cano-Renteria F., DeLacy B. G. (2018). Nanophotonic particle simulation and inverse design using artificial neural networks. *Sci. Adv.*.

[15] Liu D., Tan Y., Khoram E., Yu Z. (2018). Training deep neural networks for the inverse design of nanophotonic structures. *ACS Photonics*.

[16] Unni R., Yao K., Zheng Y. (2020). Deep convolutional mixture density network for inverse design of layered photonic structures. *ACS Photonics*.

[17] Hegde R. (2021). Sample-efficient deep learning for accelerating photonic inverse design. *OSA Contin.*.

[18] Jiang J., Fan J. A. (2021). Multiobjective and categorical global optimization of photonic structures based on ResNet generative neural networks. *Nanophotonics*.

[19] So S., Mun J., Rho J. (2019). Simultaneous inverse design of materials and structures via deep learning: demonstration of dipole resonance engineering using core–shell nanoparticles. *ACS Appl. Mater. Interfaces*.

[20] Qu Y., Jing L., Shen Y., Qiu M., Soljacic M. (2019). Migrating knowledge between physical scenarios based on artificial neural networks. *ACS Photonics*.

[21] So S., Lee D., Badloe T., Rho J. (2021). Inverse design of ultra-narrowband selective thermal emitters designed by artificial neural networks. *Opt. Mater. Express*.

[22] Ma W., Cheng F., Liu Y. (2018). Deep-learning-enabled on-demand design of chiral metamaterials. *ACS Nano*.

[23] Ma W., Cheng F., Xu Y., Wen Q., Liu Y. (2019). Probabilistic representation and inverse design of metamaterials based on a deep generative model with semi-supervised learning strategy. *Adv. Mater.*.

[24] Liu Z., Zhu D., Lee K. T., Kim A. S., Raju L., Cai W. (2020). Compounding meta-atoms into metamolecules with hybrid artificial intelligence techniques. *Adv. Mater.*.

[25] Sajedian I., Kim J., Rho J. (2019). Finding the optical properties of plasmonic structures by image processing using a combination of convolutional neural networks and recurrent neural networks. *Microsyst. Nanoeng.*.

[26] Jensen J. S., Sigmund O. (2011). Topology optimization for nano-photonics. *Laser Photonics Rev.*.

[27] Molesky S., Lin Z., Piggott A. Y., Jin W., Vucković J., Rodriguez A. W. (2018). Inverse design in nanophotonics. *Nat. Photonics*.

[28] Fan J. A. (2020). Freeform metasurface design based on topology optimization. *MRS Bull.*.

[29] Wen F., Jiang J., Fan J. A. (2020). Robust freeform metasurface design based on progressively growing generative networks. *ACS Photonics*.

[30] Zhang D., Bao Q., Chen W., Liu Z., Wei G., Xiao J. J. (2021). Inverse design of an optical film filter by a recurrent neural adjoint method: an example for a solar simulator. *J. Opt. Soc. Am. B*.

[31] Li L., Dobrowolski J. A. (2000). High-performance thin-film polarizing beam splitter operating at angles greater than the critical angle. *Appl. Opt.*.

[32] Willey R. R. (2002). *Practical Design and Production of Optical Thin Films*.

[33] Keshavarz Hedayati M., Elbahri M. (2016). Antireflective coatings: conventional stacking layers and ultrathin plasmonic metasurfaces. A mini review. *Materials*.

[34] Macleod H. A. (2010). *Thin-Film Optical Filters*.

[35] Bishop C. M. (1994). *Mixture Density Networks*.

[36] Malkiel I., Mrejen M., Nagler A., Arieli U., Wolf L., Suchowski H. (2018). Plasmonic nanostructure design and characterization via deep learning. *Light Sci. Appl.*.

[37] Deng Y., Ren S., Fan K., Malof J. M., Padilla W. J. (2021). Neural-adjoint method for the inverse design of all-dielectric metasurfaces. *Opt. Express*.

[38] Born M., Wolf E. (1999). *Principles of Optics: Electromagnetic Theory of Propagation, Interference and Diffraction of Light*.

[39] Rodríguez-de Marcos L. V., Larruquert J. I., Méndez J. A., Aznárez J. A. (2017). Self-consistent optical constants of MgF_2_, LaF_3_, and CeF_3_ films. *Opt. Mater. Express*.

[40] Rodríguez-de Marcos L. V., Larruquert J. I., Méndez J. A., Aznárez J. A. (2016). Self-consistent optical constants of SiO_2_ and Ta_2_O_5_ films. *Opt. Mater. Express*.

[41] Niederreiter H. (1988). Low-discrepancy and low-dispersion sequences. *J. Number Theor.*.

[42] Kogelnik H., Shank C. V. (1971). Stimulated emission in a periodic structure. *Appl. Phys. Lett.*.

[43] Shyh W. (1974). Principles of distributed feedback and distributed Bragg-reflector lasers. *IEEE J. Quant. Electron.*.

[44] Gao L., Lemarchand F., Lequime M. (2012). Exploitation of multiple incidences spectrometric measurements for thin film reverse engineering. *Opt. Express*.

[45] Iten R., Metger T., Wilming H., Del Rio L., Renner R. (2020). Discovering physical concepts with neural networks. *Phys. Rev. Lett.*.

[46] Liu Z., Raju L., Zhu D., Cai W. (2020). A hybrid strategy for the discovery and design of photonic structures. *IEEE J. Emerg. Sel. Topics Circuits Syst.*.

[47] Kiarashinejad Y., Zandehshahvar M., Abdollahramezani S., Hemmatyar O., Pourabolghasem R., Adibi A. (2020). Knowledge discovery in nanophotonics using geometric deep learning. *Adv. Intell. Syst.*.

[48] Kiarashinejad Y., Abdollahramezani S., Zandehshahvar M., Hemmatyar O., Adibi A. (2019). Deep learning reveals underlying physics of light–matter interactions in nanophotonic devices. *Adv. Theory Simul.*.

[49] Yeung C., Tsai J. M., King B., Kawagoe Y., Ho D., Knight M. W. (2020). Elucidating the behavior of nanophotonic structures through explainable machine learning algorithms. *ACS Photonics*.

