# Supplementary material for: A mixture-density-based tandem optimization network for on-demand inverse design of thin-film high reflectors
Source: Nanophotonics. 2021 Oct 8;10(16):4057–65. doi: 10.1515/nanoph-2021-0392 (PMC9651023; doi:10.1515/nanoph-2021-0392)
Supplement: Supplementary file 1 — Supplementary Material [file j_nanoph-2021-0392_suppl_001.docx]

**Supplementary Materials**

**A Mixture-Density-Based Tandem Optimization Network for On-Demand Inverse Design of Thin-Film High Reflectors**

Rohit Unni,^1,2^ Kan Yao,^1,2^ Xizewen Han,^3^ Mingyuan Zhou,^3,4^ and Yuebing Zheng^1,2,*^

^1^Walker Department of Mechanical Engineering, The University of Texas at Austin, Austin, Texas 78712, USA

*^2^Texas Materials Institute, The University of Texas at Austin, Austin, Texas 78712, USA*

*^3^Department of Statistics and Data Science, The University of Texas at Austin, Austin, Texas 78712, USA*

*^4^McCombs School of Business, The University of Texas at Austin, Austin, Texas 78712, USA*

^*^Corresponding Author: [zheng@austin.utexas.edu](mailto:zheng@austin.utexas.edu)

**This SM includes:**

**1. Neural Network (NN) Model Architectures and Hyperparameters**

**2. Optimization Performance**

**3. Datasets and Multimodality**

**1. Neural Network (NN) Model Architectures and Hyperparameters**

The MDN begins with a series of convolutional and pooling layers. An input with dimension 300 is fed into two consecutive one-dimensional (1D) convolution layers with 64 and 32 filters, a max pooling layer, and then another pair of 1D convolution and max pooling layers. The output is flattened and fed into 3 FC layers leading into the final mixture density layer containing 16 mixtures. Each output design variable has its own 16 mixtures, each with a mean and variance, and each of the 16 mixtures has a mixing parameter that is shared across all variables. Therefore, the output has a total of 20*16*2 + 16 = 656 dimensions. The forward model takes in the 20-dimensional input design vector and simply uses five FC layers with progressively decreasing numbers of neurons leading into the 300-dimensional output of the predicted spectra. Both model architectures are shown in Figure S1. The MDN uses the negative log-likelihood metric as its loss function, and the forward model uses RMSE. Both models are built in the open-source Python library Keras, using TensorFlow on the backend, and the Adam optimizer is used with a learning rate of 0.001 and a batch size of 100. All the models used in the main text are trained for 500 epochs, and the training curves of the models used for Figs. 2 and 3 are shown in Figure S2. For the MDN curve, the built-in loss tracker for training shows slightly higher results for training data values when using the negative log-likelihood, however an evaluation of the full training and test sets after fitting yielded the -18 loss for both cited in the main text. For all models’ datasets, design values are rescaled to the range [0, 1] for ease of training.

**2. Optimization Performance**

The post-processing method outlined in the main text features design variables optimized one at a time in several cycles. Since the relationship between the design variables and output response is non-monotonic, optimizing one variable at a time and cycling through the design vector is not guaranteed to converge towards a global optimum. In practice, however, this method tends to lead to higher performance improvements in less time than sampling all 20 variables simultaneously. A modified version of the post-processing algorithm was developed that can simultaneously sample multiple design variable distributions, with the number of variables concurrently optimized specified by the user, up to the total number of design parameters. However, this generally led to equivalent or worse performance on average compared with the single variable approach, so the latter is used for all the optimization discussed in the main text. Furthermore, the resampling of MDN’s outputs can theoretically be run infinitely long, but in practice, there are diminishing returns for performance improvement beyond a few hundred samples taken per design variable. The processing used for benchmarking the model in Figure 2 uses four rounds of sampling with 50 samples each, for a total of 200 samples taken per design variable.

To further demonstrate the advantages of the MDN post-processing, a separate post-processing algorithm using gradient descent is implemented. The training process of an NN calculates the gradient of the loss function with respect to each weight using the chain rule in order to improve itself over time. Likewise, the gradient of the loss with respect to the input, the design vector, can be directly calculated to perform inverse design. For this post-processing method, we take the same initial suggestion from the MDN, but instead of sampling for new candidate designs from the MDN distributions, the initial suggestion is fed into the forward model, and the gradient of the loss with respect to the design is calculated. This is multiplied by a learning rate and subtracted from the design vector to give a new design. The new design is then fed into the forward model again to calculate a new gradient, and the process repeats to continually optimize the design, similar with the previous post-processing method. After testing a variety of learning rates, a value of 0.001 provided the best results. A chart demonstrating the relation between samples taken and model improvement for both the MDN resampling processing and the gradient descent processing is shown in Figure S3. For each case, the ratio between response RMSE before and after post-processing is calculated to measure the performance improvement, and this is averaged across 50 random samples from the test dataset. Overall, the MDN resampling leads to higher improvement in design performance compared to the gradient-descent-based method.

**3. Datasets and Multimodality**

The data described in the main text predominantly uses data from Rodriguez-de Marcos et al for the dielectric function of Ta_2_O_5_[1]. For the designs with extended high-reflectance zones, as shown in Figure 4, an alternate data source from Gao et al is used[2]. Due to the difference in deposition methods, the latter source features less dielectric loss in the lower wavelengths and thus reduces undesired absorbance in these regions, resulting in higher reflectance. A comparison of a single design simulated using both data sources is shown in Figure S4. For the new dielectric function, a separate dataset is generated, with the same total size of samples for training and test sets. A separate pair of MDN and forward network, both with the same architectures, are trained for the same number of epochs and converge to similar loss values.

The data in the main text also features a non-uniform distribution across the design space, with 50% of the data uniformly sampling the entire thickness range, and 25% uniformly sampling each of the upper and lower halves of the ranges. This splitting is conducted to give the dataset better representation of lower variance as well as high variance designs. With 20 total design variables, the chances of a given design having all values within a certain range is quite low. This lack of designs with balanced lower variance can manifest in the dataset in a way similar to the class imbalance problem in classification machine learning tasks, that can noticeably affect model performance. To address this, the adjusted dataset featured in the main text is used. However, for comparison, a separate set of networks were created and trained on a dataset that entirely samples the thickness ranges uniformly. The dataset is generated to be the same size, and the MDN and forward model are trained with the same architecture for the same number of epochs. The model trained on the uniformly sampled data is still able to produce accurate design suggestions on random structures with its MDN, which can be further improved by post-processing with the forward model. Similar results to those in Figure 2 are shown for the uniform data model in Figure S5, using the same ground truth input. The model is also able to retrieve periodic designs of DBRs, albeit at slightly worse performances than the tandem optimization model trained on the adjusted dataset used in the main text. The difference in performance for the cases of shorter center wavelength is minor, however for cases of higher center wavelength, the imbalance in the dataset leads to poorer prediction by the forward model, which reduces the accuracy of the spectral matching, though a good agreement over the high-reflectance region is still achieved (see Figure S6).

To further test the performance of the model in relation to the uniformity of data, we generate a separate dataset using the method of low discrepancy sequences. While the previous datasets generated their uniform random numbers using python libraries’ built-in pseudo-random number generators, low discrepancy sequences offer the possibility of more evenly spaced data. The Sobol sequence is one such example of low discrepancy sequences, aiming to create a sequence that evenly subdivides each dimension into numbers of a power of 2 and then maps to points in that space using a series of matrix transformations[3]. With each set of points using progressively finer subdivisions in this transformation process, empty spaces are more likely to be sampled than spaces that already have points, ensuring a more evenly distributed dataset. Using the Sobol sequence functionality in the Python SciPy Quasi-Monte Carlo library, we sample all 20 design variables uniformly within the thickness ranges, with a total dataset size equivalent to the previous ones. A separate MDN and forward model are trained on this Sobol dataset, using the same network architectures and hyperparameters. On the whole, we find the model performs similarly to the previous uniform data model generated with pseudo-random methods, yielding the same average RMSE before and after post-processing and producing qualitatively similar outputs on test cases (Figure S5C,D).

In general, compared with DBRs, the designs featuring randomized variables show greater multimodality when their responses are fed into the MDN. This may be due to the quasi-periodic structures of the DBR’s may place implicit limits on the multimodality, and the MDN may predict a low chance of degenerate solutions and collapse its modes. Mainly, the DBR design distributions show close to zero multimodality, while for the randomized designs, on average around 1 in 5 contain at least one design distribution with significant multimodality. One example is shown in Figure S7, showing one constituent design distribution with two peaks. Sampling from those peaks separately while holding other variables fixed leads to similar output spectra despite the distance between them, indicating a degree of degeneracy in the solutions that the MDN can model.

**
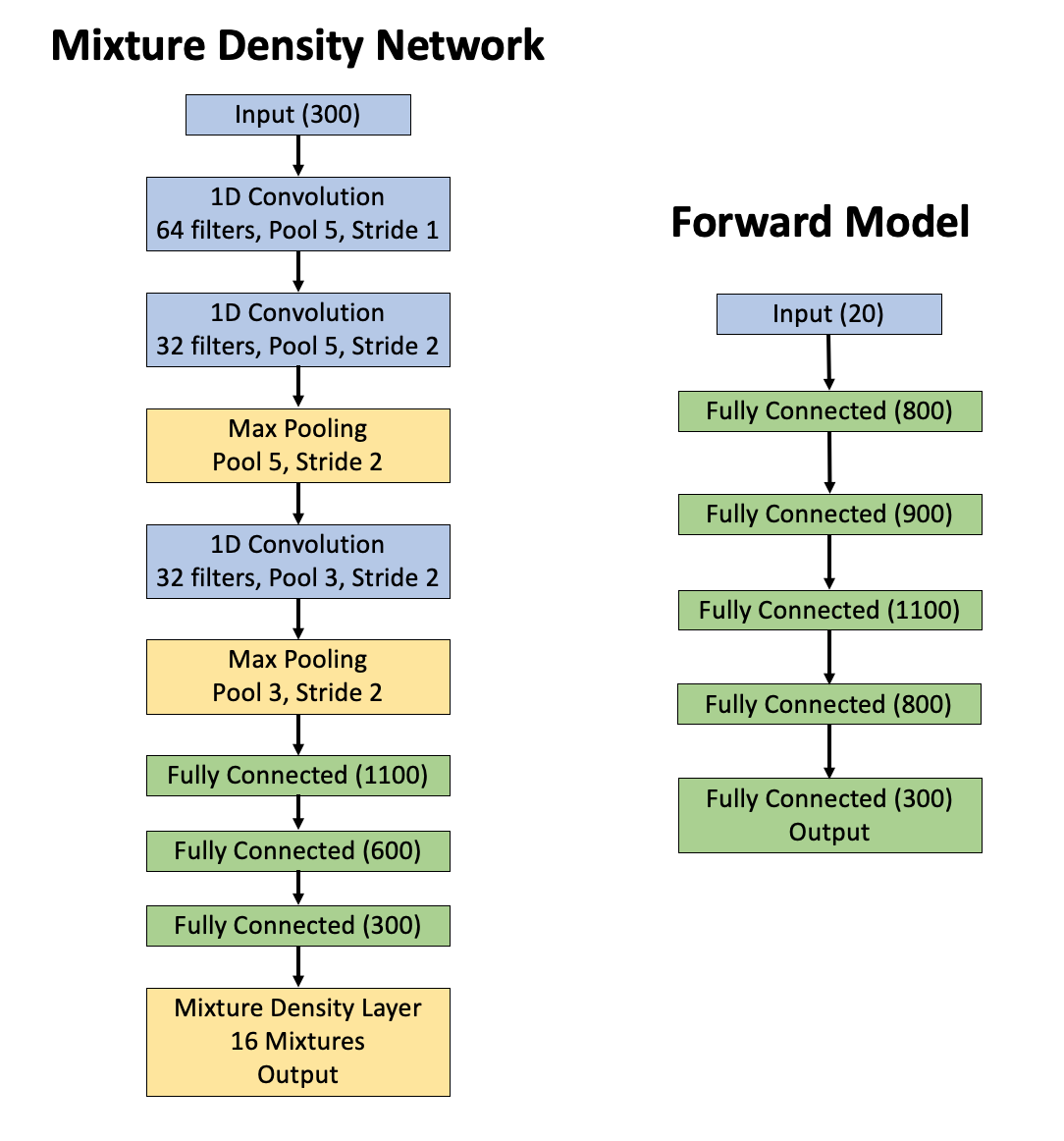
**

**Figure S1:** Visualization of model architectures used for mixture density network (Left) and the forward model (Right). Numbers in parentheses for fully connected layers represent the number of neurons in that layer.


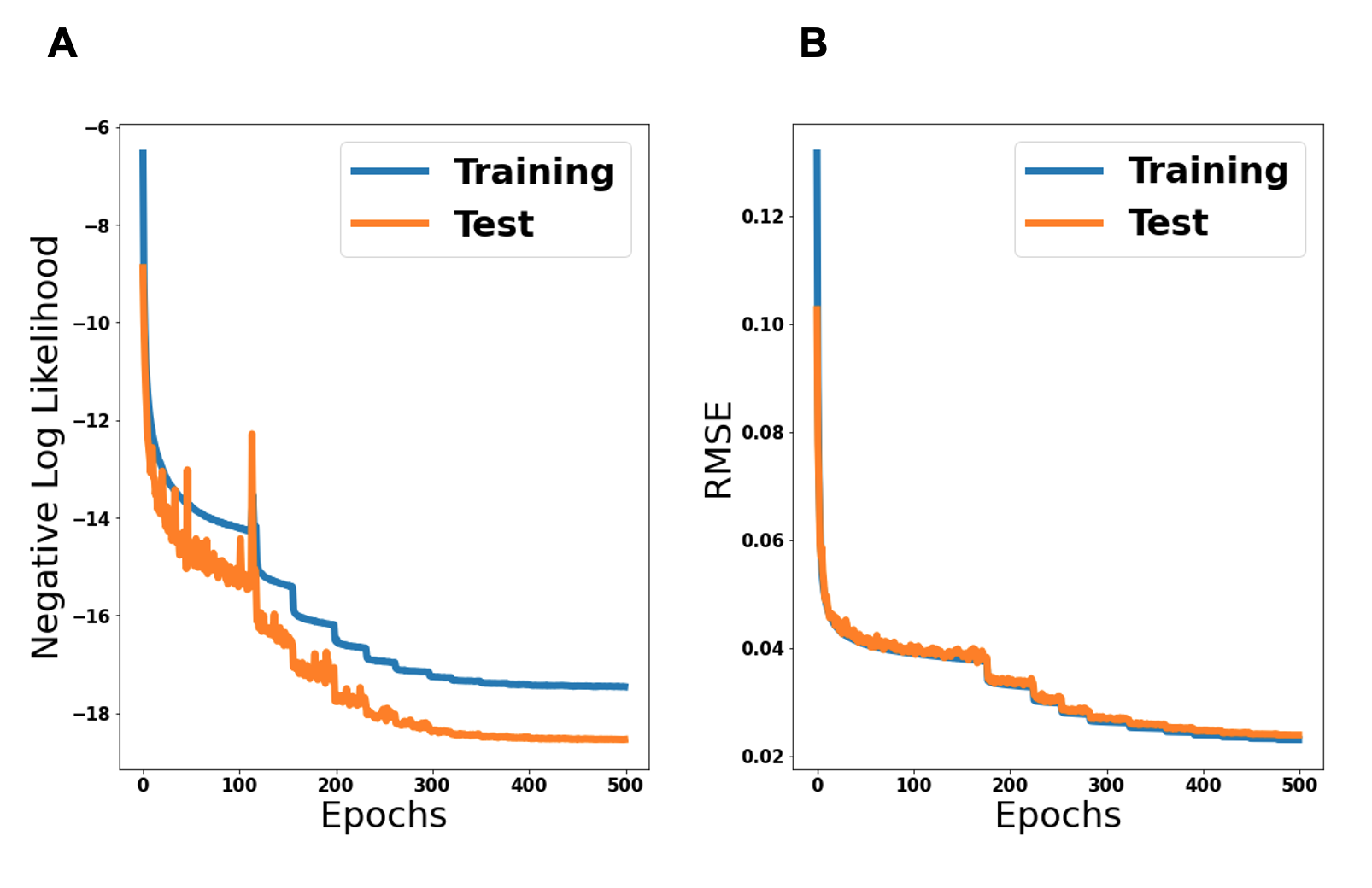


**Figure S2**: Training curve of the loss functions over 500 epochs for both training and test datasets for (**A**) the MDN model and (**B**) the forward model.

**
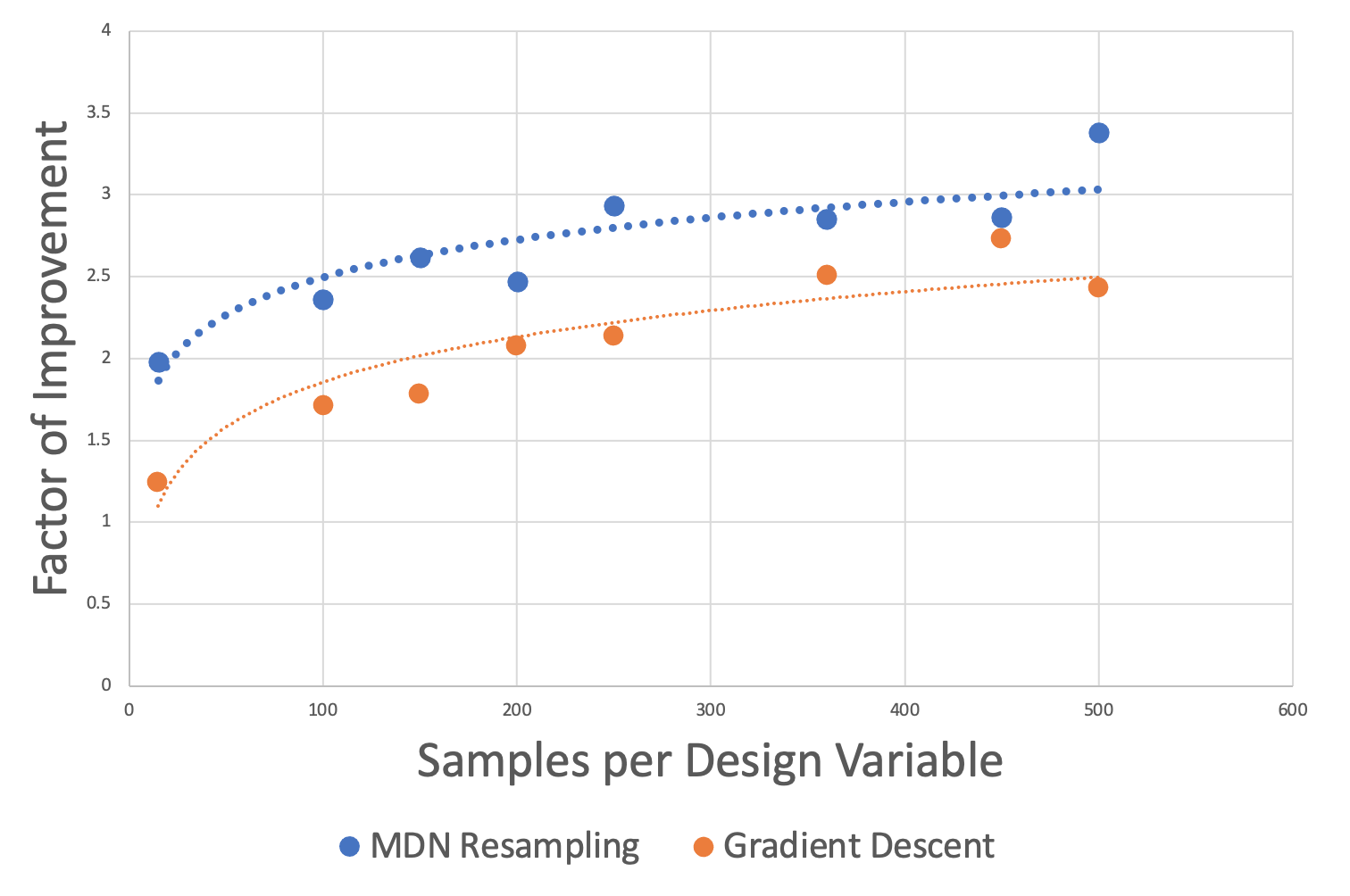
**

**Figure S3:** Relation between the factor of improvement and the total number of samples taken per design variable in the optimization, comparing the processing method using MDN resampling with one using gradient descent. The factor of improvement is defined as the ratio of RMSE before optimization to the RMSE after it. Each point is calculated as the average of 50 randomly chosen designs from the test dataset. Dotted lines indicating the trend are added to guide the eyes.


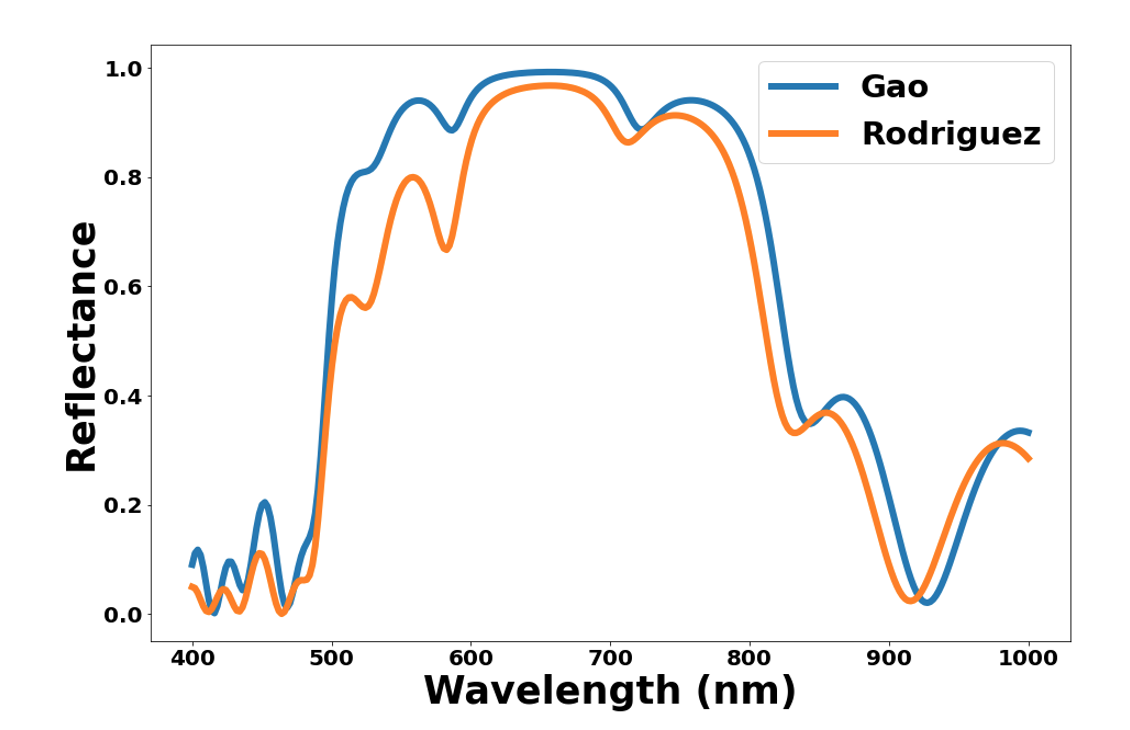


**Figure S4:** Comparison of high reflectance spectrum with extended bandwidth as simulated using data by Gao et al (blue curve) and by Rodriguez-de Marcos et al (orange curve).

**
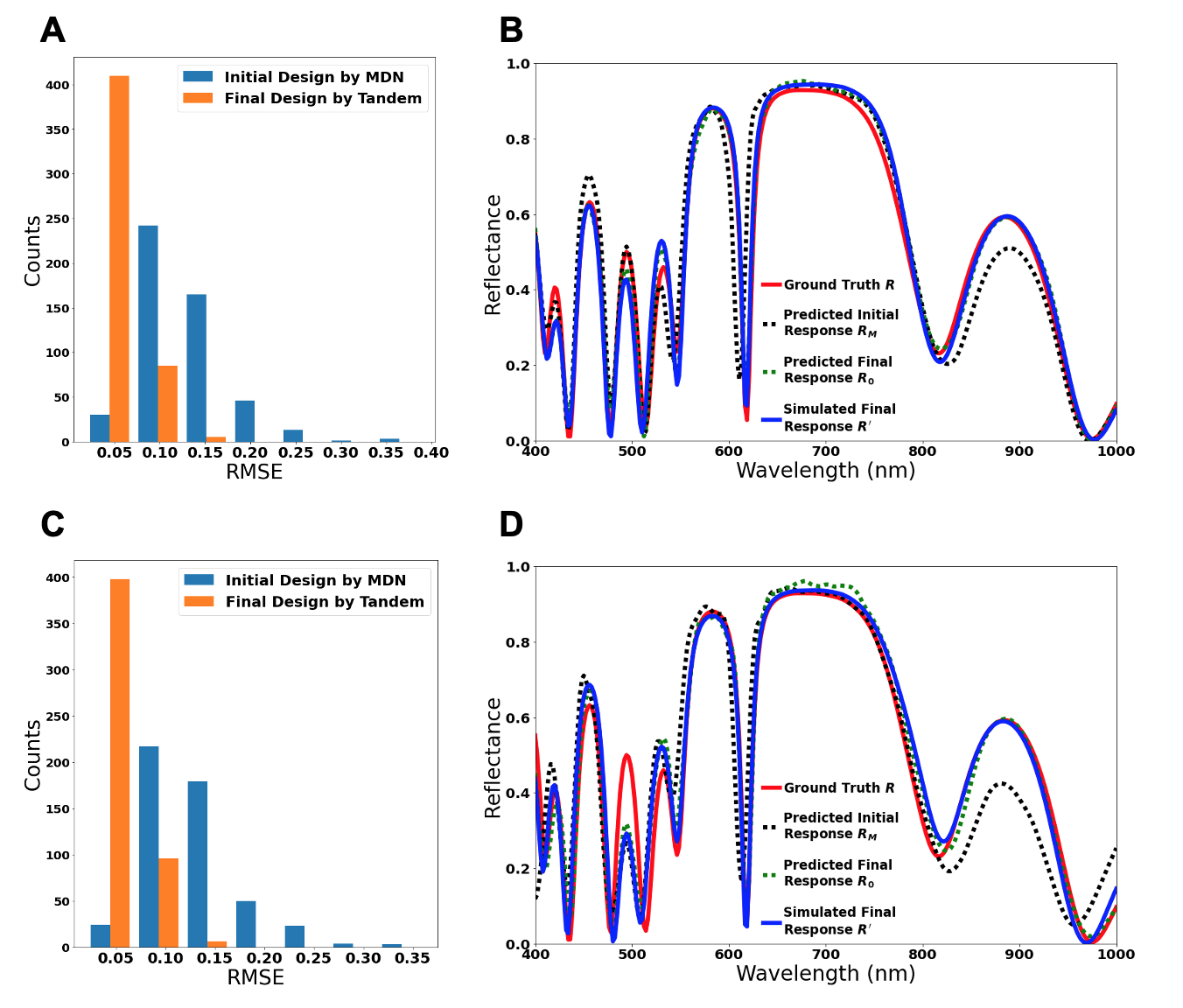
**

**Figure S5:** (**A**) Histogram of RMSE matching between the requested spectrum and the response of the model-suggested design for 500 randomly chosen test dataset samples for the ensemble model trained on the uniformly sampled data using pseudorandom generation. (**B**) Comparison of the same requested spectrum in Figure 2 (red curve) with the simulated response of the final design retrieved by the model (blue curve), and with the forward model predicted responses for the initial design (black curve) and final design (green curve), all calculated using uniform data model. (**C**) Histogram of RMSE matching for 500 randomly chosen test dataset samples for the model trained on the uniform dataset generated with Sobol sequences. (**D**) Comparison of the same requested spectrum as in (**B**) using the Sobol-sequence-trained model.

**
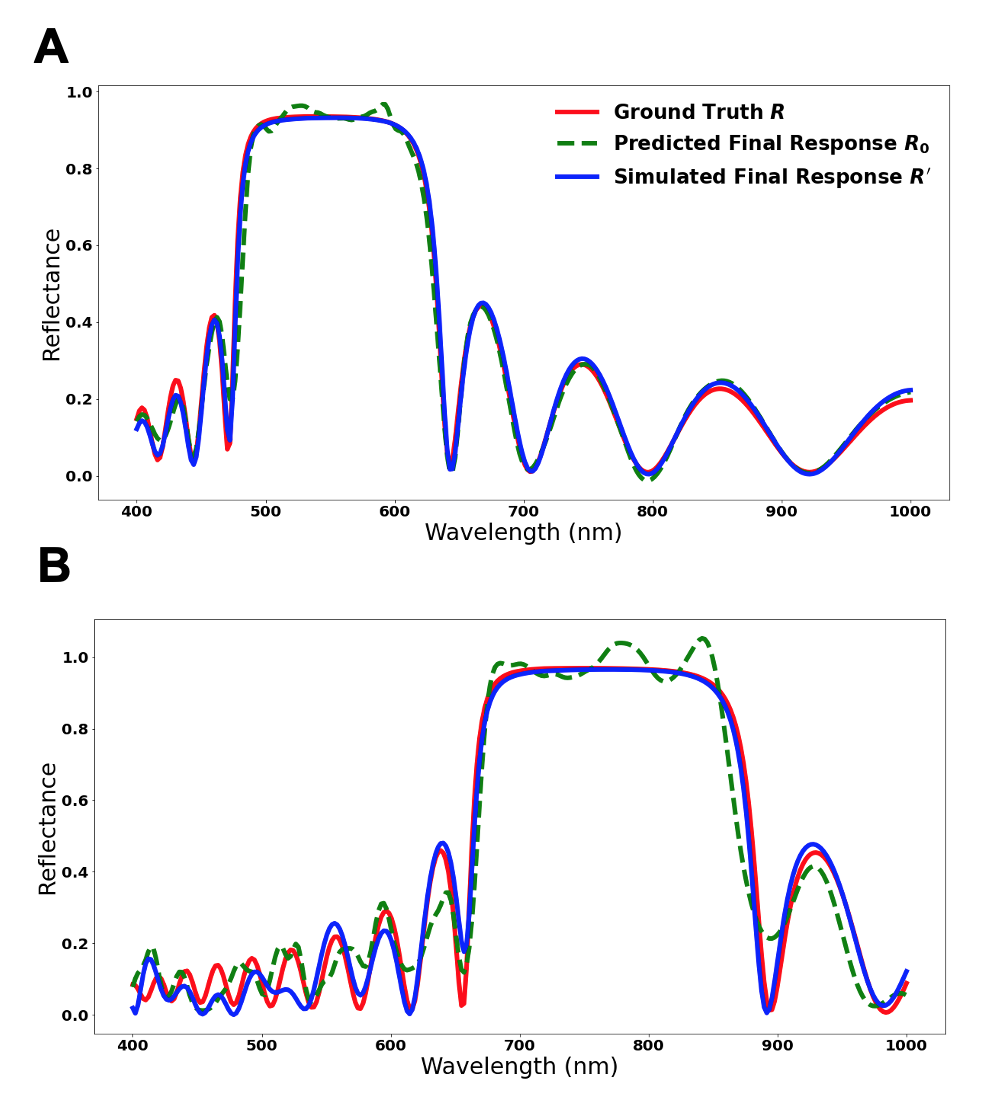
**

**Figure S6:** Two examples showing the comparison of the requested spectrum from a DBR (red curve) with the simulated response of a design retrieved by the mode (blue curve). Predicted response of the design by the forward network (green dash curve) is also shown for comparison. The results are obtained using the uniform data model.


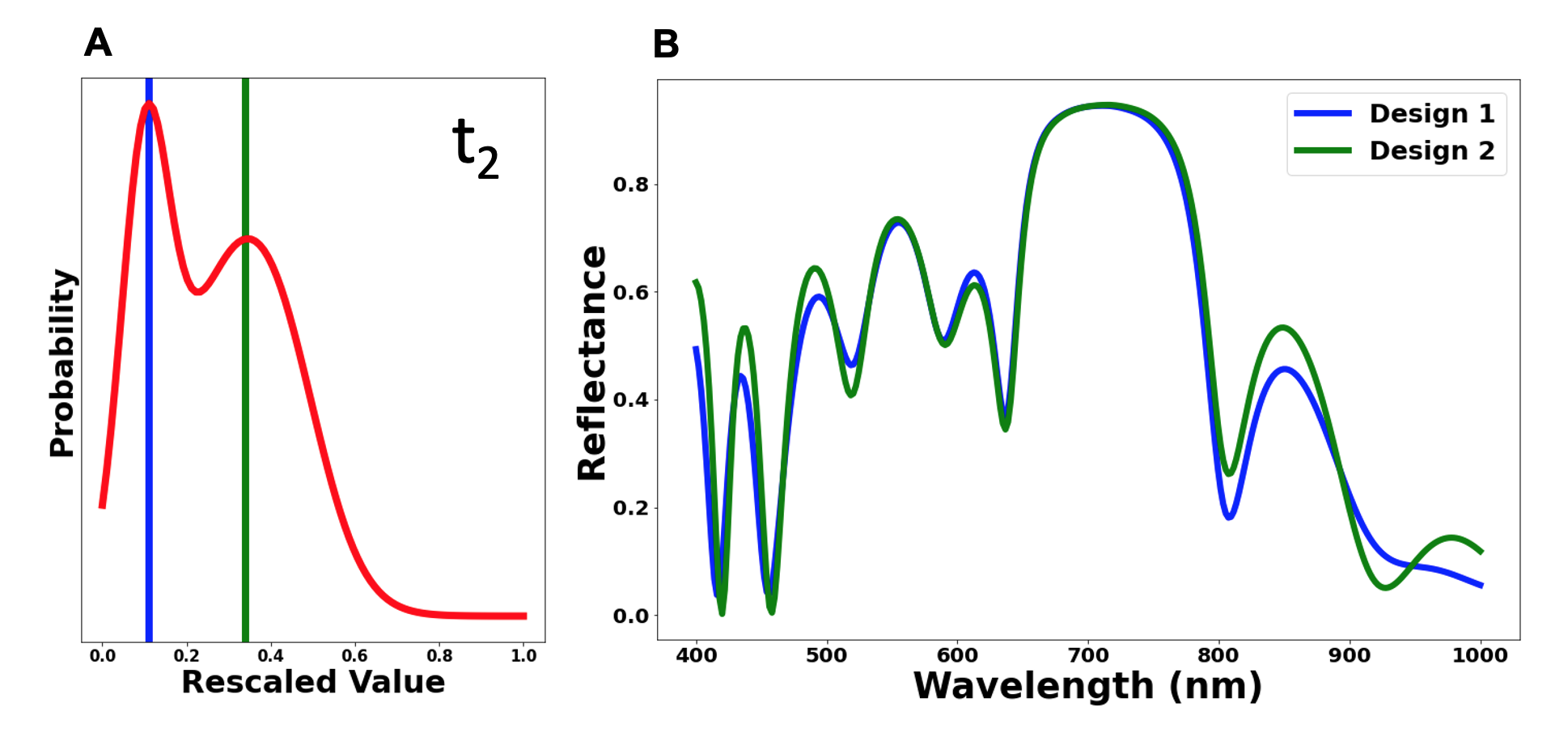


**Figure S7:** (**A**) Design distribution produced by the MDN for the second layer of a randomized 20-layer thin-film structure. Samples are taken at two prominent peaks denoted by the blue and green lines. (**B**) Comparison of spectra produced by designs using the sampled thicknesses in (**A**) for layer 2, with all other layer thicknesses fixed.

**References**

1. Rodríguez-de Marcos, L.V., et al., *Self-consistent optical constants of SiO2 and Ta2O5 films.* Optical Materials Express, 2016. **6**(11): p. 3622-3637.

2. Gao, L., F. Lemarchand, and M. Lequime, *Exploitation of multiple incidences spectrometric measurements for thin film reverse engineering.* Optics Express, 2012. **20**(14): p. 15734-15751.

3. Niederreiter, H., *Low-discrepancy and low-dispersion sequences.* Journal of Number Theory, 1988. **30**(1): p. 51-70.
